# In Search of Biomarkers for Autism Spectrum Disorder

**DOI:** 10.1002/aur.2026

**Published:** 2018-10-15

**Authors:** Marta Del Valle Rubido, James T. McCracken, Eric Hollander, Frederick Shic, Jana Noeldeke, Lauren Boak, Omar Khwaja, Shamil Sadikhov, Paulo Fontoura, Daniel Umbricht

**Affiliations:** ^1^ Roche Innovation Center Basel Roche Pharmaceutical Research and Early Development NORD Basel Switzerland; ^2^ Psychiatry and Behavioral Sciences David Geffen School of Medicine at UCLA Los Angeles California; ^3^ Psychiatry and Behavioral Sciences Albert Einstein College of Medicine and Montefiore Medical Center Bronx New York; ^4^ Center for Child Health, Behavior and Development Seattle Children's Research Institute Seattle Washington; ^5^ Department of Pediatrics University of Washington Seattle Washington; ^6^ Roche Product Development Neuroscience Basel Switzerland

**Keywords:** biomarker, eye movement, olfactory, social cognition

## Abstract

Autism Spectrum Disorder (ASD) lacks validated measures of core social functions across development stages suitable for clinical trials. We assessed the concurrent validity between ASD clinical measures and putative biomarkers of core deficits, and their feasibility of implementation in human studies. Datasets from two adult ASD studies were combined (observational study [*n* = 19] and interventional study baseline data [*n* = 19]). Potential biomarkers included eye‐tracking, olfaction, and auditory and visual emotion recognition assessed *via* the Affective Speech Recognition test (ASR) and Reading‐the‐Mind‐in‐the‐Eyes Test (RMET). Current functioning was assessed with intelligence quotient (IQ), adaptive skill testing, and behavioral ratings. Autism severity was determined by the Autism Diagnostic Observation Scale‐2 and Social Communication Interaction Test (SCIT). Exploratory measures showed varying significant associations across ASD severity, adaptive skills, and behavior. Eye tracking endpoints showed little relationship to adaptive ability but correlated with severity and behavior. ASR scores significantly correlated with most adaptive behavior domains, as well as severity. Olfaction predicted visual and auditory emotion recognition. SCIT scores related moderately to multiple severity domains, and was the only measure not related with IQ. RMET accuracy was less related to ASD features. Eye tracking, SCIT, and ASR showed high test–retest reliability. We documented associations of proximal biomarkers of social functioning with multiple ASD dimensions. With the exception of SCIT, most correlations were modest, limiting utility as proxy measures of social communication. Feasibility and reliability were high for eye‐tracking, ASR, and SCIT. Overall, several novel experimental paradigms showed potential as social biomarkers or surrogate markers in ASD. ***Autism Research** 2018, 11: 1567–1579*. © 2018 The Authors. Autism Research published by International Society for Autism Research and Wiley Periodicals, Inc.

**Lay Summary:**

More accurate measurements of treatment effects are needed to help the development of new drug treatments for autism spectrum disorders (ASD). This study evaluates the relationship between assessments designed to measure behaviors associated with social communication and cognition in ASD with clinical and diagnostic assessments of symptom severity as well as their implementation. The assessments including eye‐tracking, auditory and visual social stimuli recognition, and olfaction identification showed potential for use in the evaluation of treatments for social difficulties in ASD.

## Introduction

Autism Spectrum Disorder (ASD) is a heterogeneous neurodevelopmental disorder with an estimated prevalence of 1 in 59 children, according to CDC's Autism and Developmental Disabilities Monitoring Network [Baio et al., 2018], or one in 132 persons worldwide [Baxter et al., 2015]. The core symptoms of ASD include impairments in social communication and interaction, restricted or repetitive behavior, and unusual sensory sensitivity or interests [American Psychiatric Association, 2013]. Common associated symptoms vary from aggression, self‐injurious behavior, impulsivity and irritability to hyperactivity, anxiety and mood symptoms [Baird, Cass, & Slonims, 2003]. Current primary treatment emphasizes forms of behavioral interventions (e.g., applied behavior analysis) to advance development and adaptive skills, but also various pharmacological treatments to target maladaptive co‐occurring conditions (psychostimulants, alpha agonists, antidepressants, and antipsychotics) [Zwaigenbaum et al., 2015]. To date, no efficacious pharmacotherapy for the core symptoms of ASD exists [Ji & Findling, 2015].

Drug development for core impairments in the social and communicative domains has been limited, in part due to a lack of well validated, sensitive measures suitable for clinical trials across the life span [Anagnostou et al., 2015; Baxter et al., 2015; Brugha, Doos, Tempier, Einfeld, & Howlin, 2015; Zwaigenbaum et al., 2015; Zwaigenbaum, Bryson, & Garon, 2013], in contrast to restricted interests, repetitive behaviors, and anxiety, where such measures are available [Lecavalier et al., 2014; Scahill et al., 2015]. There are even larger gaps in the development of valid core symptom outcome measures and biomarkers (defined as “a characteristic that is objectively measured and evaluated as an indicator of normal biological processes, pathogenic processes or pharmacologic responses to a therapeutic intervention” [Strimbu & Tavel, 2010]), for adults with ASD [Brugha et al., 2015]. Most of the evidence in the research of clinical endpoints in ASD originates from studies in young children. This is suboptimal for the purpose of drug development, where the ICH guidelines [European Medicines Agency, 2000] recommend the start of pediatric interventional studies after substantial experience in adults for drugs in development for non‐serious and non‐life‐threatening indications.

Treatment development in ASD is also challenged by the phenotypic and etiologic heterogeneity of the disorder [Geschwind & Levitt, 2007], including the evidence that the core symptom dimensions may have separate genetic architectures [Ronald et al., 2006], which hinders the identification of drug targets, compared with other single gene disorders (e.g., mammalian Target Of Rapamycin (mTOR) pathway in tuberous sclerosis). However, all individuals with ASD share social impairments in relatedness and reciprocity and communication deficits, argued to represent a convergence of etiologies in terms of shared neurobiology [Happe & Ronald, 2008]. Research aiming to delineate disruptions in biological processes has spurred considerable study of the cognitive phenotypes of ASD. Not surprisingly, efforts to identify the major cognitive contributors to social impairments in ASD have revealed a multifaceted underpinning of these core processes and deficits [Adolphs, 2001].

Diagnostic scales used in ASD target relatively heterogeneous groups of behaviors and were not originally developed to sensitively assess social communication or more narrow components of social responsiveness in the context of a clinical trial. By understanding the component processes underlying social cognition and communication, therapeutic effects should be more easily identified and quantified more accurately. Results from contemporary investigations attempting to fractionate social and communication impairments in ASD and link them mechanistically to biologically proximal information processing functions have been mixed; no single biomarker or cognitive domain has emerged as “primary” thus far. Studies find considerable overlap in performance between ASD individuals and controls, and more variability and likely subgroups within ASD subjects, such as for facial emotional recognition [Jones & Klin, 2013], supporting phenotypic and genetic heterogeneity. Therefore, there is a great need for the identification of biomarkers, or objective indicators, of deficits at different system levels of social cognition and social communication in ASD which can mechanistically be related to symptoms—moving from proximal to distal levels of information processing and integration to behavior—and are sensitive to change, and may therefore be used as reliable outcome measures and stratification of patient populations in treatment trials [Beversdorf & Missouri Autism Summit, 2016; Jeste, Frolich, & Loo, 2015; Strimbu & Tavel, 2010]. Charting behavioral changes arising from pharmacological effects on pathophysiology and disease processes with valid and precise assessments will facilitate the development of more efficacious, targeted treatments in ASD [Jeste et al., 2015; Zwaigenbaum et al., 2013]. As the initial evaluation of novel compounds usually takes place in adult patients who may differ in many aspects from the treatment population of children, it is necessary to establish biomarkers not only in children or adolescents, but also for adults.

The primary objective of this study was to assess the concurrent validity of exploratory assessments of social information processing and cognition in adult patients with ASD through characterization of their relationship with standardized measures of symptoms, behavior, and functioning. These measures included eye‐tracking paradigms as a measure of attunement to, and extraction of, socially relevant information, and Affective Speech Recognition test (ASR), and Reading‐the‐Mind‐in‐the‐Eyes Test (RMET), as measures of the ability to detect and process socially relevant information in human communication. We also explored effects on olfaction as a sensory modality assumed to play a role in social interaction, and a novel clinical assessment, the Social Communication Interaction Test (SCIT), to directly evaluate separate domains of social communication. The second objective was to examine the feasibility to implement these exploratory assessments in a clinical study context across multiple sites in order to gauge potential application in clinical trials. A companion manuscript describing the assessment of the discriminant validity of these exploratory assessments between ASD and healthy controls is currently in preparation.

## Methodology

### Design

**Figure 1:**
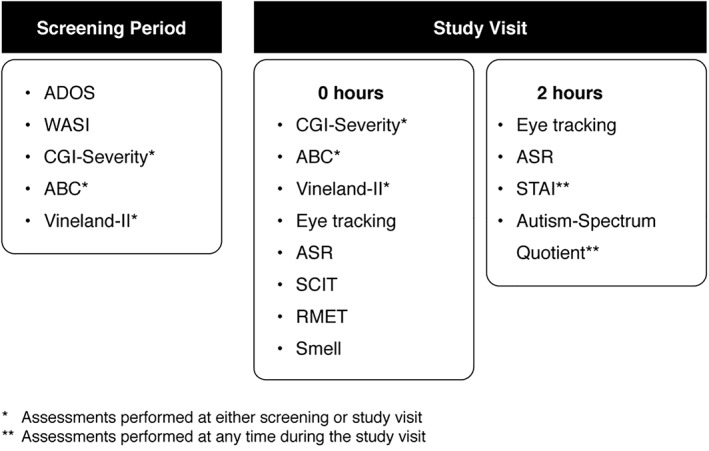
Study design and schedule of assessments. Autism‐Spectrum Quotient results are not addressed in this manuscript. ABC: aberrant behavior checklist; ADOS: autism diagnostic observation schedule; ASR: affective speech recognition; CGI: clinical global impression; RMET: reading the mind in the eyes test; SCIT: social communication interaction test; STAI: state/trait anxiety inventory; WASI: Wechsler abbreviated scale of intelligence.

The data for this report are derived from two studies conducted at the same three academic centers in the United States: (a) A multicenter, observational study (http://clinicaltrials.gov: NCT01669889); and (b) an interventional study [http://clinicaltrials.gov: NCT01474278; Umbricht et al., 2017], for which the data included in this analysis were collected at baseline, prior to any drug administration. In both studies, identical exploratory assessments were administered in the same order. Study 1 included a healthy volunteer arm, which is not included in the current analysis (Fig. 1).

With the definition of a biomarker in mind, the clinical assessments used in this study were selected in order to provide an evaluation of different system levels of social cognition and communication. Assessing from a basic level of screening and acquiring socially relevant information (eye tracking and olfaction), to intermediate levels corresponding to the ability to capture composite information that is critical for social communication (ASR, RMET) which would finally be mirrored in behavioral aspects of social communication (ABC, scripted interaction). This multi‐dimensional approach to social communication in ASD allows for the identification of the measures that best relate to neurobiological or neurocognitive processes and to the disease and/or symptom severity. The olfaction test was selected because of its involvement in the vasopressin system in the context of the development of the vasopressin antagonist RG7713 in the Phase 1 clinical study NCT01474278 [Umbricht et al., 2017]. There is evidence of high expression of V1a receptor (V1aR) transcript in the ventral and lateral portion of the anterior olfactory nucleus, different structures of olfactory bulb and an olfactory (piriform) cortex and presence of V1AR mRNA in endothelial cells of midline blood vessels between the main olfactory bulbs in rats [Ostrowski et al., 1994].

For Study 1, the clinical and functional measures were conducted at the screening visit. For Study 2, data were taken from the baseline period only, prior to the administration of any intervention (Fig. 1). The baseline clinical evaluation consisted of the administration of the Autism Diagnostic Observation Schedule (ADOS) [Lord, Rutter, DiLavore, & Risi, 2002], Wechsler Abbreviated Scale of Intelligence‐Second Edition [Wechsler, 2008], Aberrant Behavior Checklist (ABC) [Aman, Singh, Stewart, & Field, 1985], Vineland Adaptive Behavior Scales‐Second Edition [Sparrow, 2011], Clinical Global Impression‐Severity scale (CGI‐S) [Guy, 1976], and State–Trait Anxiety Inventory (STAI) [Spielberger, 2010] (a full description of these scales is included in the Supporting Information).

### Inclusion Criteria

High‐functioning (intelligence quotient [IQ] > 70) male individuals (18–45 years of age) diagnosed with autistic disorder according to the Diagnostic and Statistical Manual of Mental Disorders, 4th edition, confirmed by clinical evaluation, were recruited in both studies. Concomitant medications were allowed as long as they had been stable for 4 weeks prior to screening and the adjustment of the therapies throughout the study was not permitted. Full inclusion and exclusion criteria are presented in Supporting Information Table S1.

### Exploratory Assessments

#### Eye tracking and pupillometry

A Tobii T60XL 60 Hz eye‐tracking system was used to continuously measure gaze position and pupil size during the following paradigms (a full description of these paradigms is included in the Supporting Information): (a) activity monitoring [based on Shic, Bradshaw, Klin, Scassellati, & Chawarska, 2011; Shic et al., 2014]; (b) biological motion preference (biomotion) [based on Annaz, Campbell, Coleman, Milne, & Swettenham, 2012]; (c) biological motion detection (biodetection) [Kaiser, Delmolino, Tanaka, & Shiffrar, 2010]; (d) complex social tasks [based on Klin, Jones, Schultz, Volkmar, & Cohen, 2002]; (e) gaze and (f) gender discrimination in a static face‐scanning task [Andari et al., 2010]; and (g) human activity preference task (human activity or social *versus* geometric) [Pierce, Conant, Hazin, Stoner, & Desmond, 2011]. These tasks were selected to span multiple domains associated with social cognition. For instance, complex social tasks are arguably the closest analogue to passive observations of real‐world human interactions. By contrast, biological motion detection and preference tasks are designed to target elementary recognition of biological signals—an evolutionarily preserved and early developing skill some researchers consider as fundamental index of social cognition [Simion, Regolin, & Bulf, 2008; Vallortigara, Regolin, & Marconato, 2005].

The ratios of time spent looking at predefined areas or areas of interest (AOI) (e.g., head, body, and background) *versus* total amount of time looking at the whole screen for each paradigm were the key outcome measures. An *a priori*‐defined composite score was computed (presented in the Supporting Information), which was derived from key parameters of all administered tasks, weighted toward complex social scenes (Who's Afraid of Virginia Woolf [WAVW]) with slightly less emphasis placed on biodetection and human activity preference. In this study, pupillometry refers to the study of the diameter of the eye as a function of task and not the relative pupil size changes nor the manipulation of the brightness of the presented images to evoke pupillary light reflex [as in Nyström et al., 2018].

#### Olfactory identification measure “Sniffin’ Sticks”

“Sniffin’ Sticks” [Hummel, Kobal, Gudziol, & MacKay‐Sim, 2007; Kobal et al., 1996] are 12 pen‐like devices containing different odors. Participants are required to select the correct odors from a selection of four different responses. Points are assigned for correct answers (maximum score 12); impairment is conventionally defined as a score of <10 correct answers. Subjects were instructed not to eat, to drink only water and to avoid chewing gum or using cigarettes at least 15 min before the test. A brief history was collected regarding the patient's olfactory experience as well as current allergies and nasal congestion in order to ensure validity in the test results. All subjects were considered evaluable by the investigators at the time of testing.

#### Affective speech recognition

The ASR test [Hollander et al., 2007] is a measure of emotion recognition in ASD (processing of auditory social information). Participants are played an audio recording of four sentences of neutral content with eight different emotional intonations (angry, disgusted, fearful, happy, lustful, neutral, sad, and surprised). Each emotional intonation is repeated 6 times for a total of 48 sentences. Participants must select the correct emotion from a list. Higher scores indicate better emotion recognition.

#### Reading the eye in the mind test‐revised

The RMET [Baron‐Cohen, Jolliffe, Mortimore, & Robertson, 1997] is a measure of theory of mind (TOM) and social sensitivity that involves presenting participants with 36 different pictures of the eye region only of human faces. The participant must choose one of four different emotions that describe the emotion the person is feeling [Baron‐Cohen, Wheelwright, Hill, Raste, & Plumb, 2001]. Higher scores indicate greater TOM and social sensitivity.

#### Social communication interaction test

One of authors of the present analysis (J.T.M.) has developed the SCIT, a potential objective measure of change in social communication during short‐term treatment. The SCIT is a semi‐structured interview administered and scored by a clinician trained in ASD diagnosis. It has six subscales rated on a 1–5 scale: social awareness and responsivity to the other (verbal); social awareness of and responsivity to the other (nonverbal); initiations of communication; conversational turn‐taking; appropriateness to interaction; and emotional insight. At the end of the interview, the social and interactional skills are scored from 1 (behavior is absent, deficient, odd) to 5 (typical for age) for each of the six domains; higher scores correspond to better social communication and interaction.

Subjects were asked to provide consent to be videotaped on their on‐site SCIT interviews in order for these to be sent to a single central expert reviewer, blind to all prior scores and clinical information, for an independent rating.

### Statistical Analysis

Statistical analyses were conducted with SAS software (SAS Institute Inc., Carey, North Carolina) and R (R Foundation for Statistical Computing, Vienna, Austria). Coefficients of correlation (i.e., Spearman's *ρ*) and corresponding *P*‐values were estimated between the key endpoints derived from the exploratory assessments and those from standard assessments of clinical symptomatology and functioning.

To evaluate the feasibility of implementation and reliability, measurements from eye tracking and ASR tasks that were carried out twice during the observational study, underwent a mixed‐effects analysis of variance model to estimate within‐subject and between‐subject variability (reported in terms of intra‐class coefficient of correlation [ICC]). Inter‐rater reliability of SCIT total scores was examined by calculation of Spearman correlation and corresponding *P*‐values. Family‐wise false positive error rate was controlled at a nominal 5% using a Bonferroni correction adjusting for all estimated pair‐wise correlations. The adjusted significance level is *P* < 0.00011.

### Ethics

Included studies were conducted in accordance with the principles of the Declaration of Helsinki and Good Clinical Practice at the Albert Einstein College of Medicine, Bronx, New York; UCLA Semel Institute, Los Angeles, California; and the Child Study Center, Yale University School of Medicine, New Haven, Connecticut. Study protocols were reviewed and approved by their respective institutional review boards.

## Results

The study pooled data from two ASD samples (*N* = 38), all male adults aged 18–40 years old. About 87% of the subjects (*N* = 33) were between 18 and 29 years of age.

The baseline characteristics of these two groups are reported in Table 1 and in Supporting Information Table S2. The prescription pattern of psychotropic medications at baseline is presented in the Supporting Information.

**Table 1:** Selected Baseline Characteristics of ASD Participants in Studies 1 and 2

		ASD Mean (*SD*)	Result interpretation	
Variable		*N* = 38	Scoring ranges	
Age in years		24.2 (5.8)	18–40	–
Male		38	–	–
WASI	Full‐scale IQ	101.1 (14.3)	Normal range: mean = 100, *SD* = 15	Higher scores, better skills/milder symptoms
	Verbal IQ	100.5 (16.1)		
	Performance IQ	100.6 (12.7)		
Vineland‐II	Adaptive behavior composite	63.8 (11.6)	Normal range: mean = 100, *SD* = 15	
	Communication	64.4 (18.1)		
	Daily living skills	68.7 (11.9)		
	Socialization	64.6 (13.08)		
ADOS Module 4	Total	12 (3.8)	0–32	Higher scores, worse skills/more severe symptoms
	Communication	3.0 (1.3)	0–8 (ASD cut‐off = 2)	
	Social interaction	6.7 (2.1)	0–14 (ASD cut‐off = 4)	
	Communication and social interaction	9.6 (3.0)	0–22 (ASD cut‐off = 7)	
ABC	Total	32.9 (20.9)	0–174	
	Irritability	5.9 (6.5)	0–45	
	Lethargy/social withdrawal	11.5 (7.4)	0–48	
	Stereotypic behavior	3.6 (3.6)	0–21	
	Hyperactivity	9.0 (8.1)	0–48	
	Inappropriate speech	2.9 (2.7)	0–12	
STAI		38.7 (13.5)	20–80	
CGI‐Severity		4.1 (0.6) 4 = moderately ill	1 = normal, not at all ill to 7 = among the most extremely ill	

ABC: aberrant behavior checklist; ADOS: autism diagnostic observation schedule; ASD: autism spectrum disorder; CGI: clinical global impression; STAI: state/trait anxiety inventory; WASI: Wechsler abbreviated scale of intelligence.

### Correlations between Exploratory Cognitive and Standard Clinical Measures in the ASD Cohort

**Figure 2:**
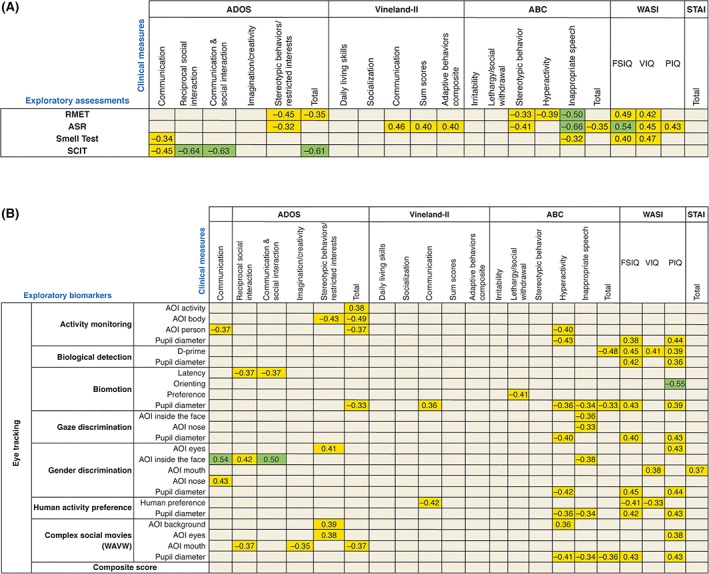
Correlations between the exploratory assessments (RMET, ASR, Smell Test, and SCIT [A]; eye‐tracking [B]) and clinical assessments. Color‐coding applied is based on the strength of the correlation coefficient: green for *r* ≥ |0.5|, orange for |0.3| < *r* < |0.5|, and gray for *r* ≤ |0.3|. All the correlations displayed are (*P* < 0.05). Correlations with *P* > 0.05 are not displayed in this Figure. *P* values <0.00011 are considered statistically significant after multiplicity adjustment. ABC: aberrant behavior checklist; ADOS: autism diagnostic observation schedule; AOI: area of interest; ASR: affective speech recognition; RMET: reading the mind in the eyes test; SCIT: social communication interaction test; STAI: state/trait anxiety inventory; WASI: Wechsler abbreviated scale of intelligence; WAVW: Who's Afraid of Virginia Woolf.

Figure 2 shows correlations between the exploratory and clinical assessments.

### Eye Tracking

Eye‐tracking measures obtained in the activity monitoring, WAVW, gender discrimination, and biological motion detection tasks predicted several ADOS domains with modest correlations (|0.35| < *r* < |0.54|). The composite score was not significantly related to the ADOS measures.

In the activity‐monitoring tasks, more time spent looking at the person was associated with lower ADOS total and ADOS communication scores (both *r* = −0.37; *N* = 36; *P <* 0.05). Interestingly, these results seemed to be driven by attention to the bodies, which was associated with fewer symptoms (ADOS total score: *r* = −0.49; *N* = 36; *P* < 0.005 and ADOS stereotypic behaviors and restricted interests: *r* = −0.43; *N* = 36; *P* < 0.01). Conversely, more time spent looking at activities was associated with higher ADOS total scores (*r* = 0.38; *N* = 36; *P* < 0.05).

In the WAVW task, more time spent looking at the mouth was associated with lower ADOS reciprocal social interaction scores (*r* = −0.37; *N* = 36; *P* < 0.05), lower ADOS imagination scores (*r* = −0.35; *N* = 36; *P* < 0.05), and lower ADOS total scores, that is, fewer deficits (*r* = −0.37; *N* = 36; *P* < 0.05). In contrast, more attention to the background was related to higher scores on stereotyped behaviors and restricted interests domains in ADOS (*r* = 0.39; *N* = 36; *P* < 0.05). Surprisingly, time looking at eyes was associated with higher ADOS stereotyped behaviors and restricted interests scores (greater severity: *r* = 0.38; *N* = 36; *P* < 0.05). During the gender discrimination task, looking inside of the face was associated with higher severity of deficits in different ADOS domains (communication: *r* = 0.54; *N* = 36; *P* = 0.001; reciprocal social interaction: *r* = 0.42; *N* = 36; *P* < 0.05; and the combination of domains communication and reciprocal social interaction: *r* = 0.50; *N* = 36; *P* < 0.005).

Replicating the relationships seen with the ADOS, on the WAVW task [Klin et al., 2002], more time spent looking at the mouth was associated with lower severity on SCIT ratings (*r* = 0.34; *N* = 36; *P* < 0.05). More time looking at the head was associated with higher RMET performance in both WAVW (*r* = 0.40; *N* = 36; *P* < 0.05) and activity monitoring tasks (*r* = 0.40; *N* = 36; *P* < 0.05). More time spent looking at the person during activity monitoring was associated with better RMET performance (*r* = 0.44; *N* = 36; *P* < 0.01). Conversely, more time spent looking at activities was correlated with lower RMET performance (*r* = −0.52; *N* = 36; *P* = 0.001).

Across all eye‐tracking paradigms, results showed no relationships to adaptive functioning (VABS). Although all correlations with behavior were modest (Table 2), several plausible significant associations with behavioral ratings were observed. Better biological motion detection (i.e., d‐prime) correlated with lower total ABC scores (*r* = −0.48; *N* = 35; *P* < 0.005). Higher biological motion preference was associated with lower scores on ABC lethargy/social withdrawal subscale (*r* = −0.41; *N* = 36; *P* < 0.05). Time looking at the mouth in the gender discrimination task was correlated with lower STAI totals (*r* = 0.37; *N* = 36; *P* < 0.05). In WAVW, attention to the background was related to higher scores on hyperactivity/noncompliance score of the ABC (*r* = 0.36; *N* = 36; *P* < 0.05). The opposite relationship with hyperactivity/noncompliance was observed in more time spent looking at the person in activity monitoring (*r* = −0.40; *N* = 36; *P* < 0.05). Moreover, looking inside of the face was associated with fewer symptoms measured with the inappropriate speech subscale of the ABC during both the gender and gaze discrimination tasks (gender discrimination: *r* = −0.38; *N* = 36; *P* < 0.05; gaze discrimination: *r* = −0.36; *N* = 36; *P* < 0.05). In addition, several significant associations between eye tracking endpoints of biological detection and human activity preference tasks were correlated with IQ. Time looking at the mouth in the gender discrimination tasks was associated with higher verbal IQ (VIQ; *r* = 0.38; *N* = 36; *P* < 0.05). Preference for social over geometric shapes in the human activity preference task was associated with lower scores on the full‐scale IQ (FSIQ; *r* = −0.41; *N* = 36; *P* < 0.05) and VIQ (*r* = −0.33; *N* = 36; *P* < 0.05).

**Table 2:** Test–Retest Reliability Data for Exploratory Assessments

Test	Item	ICC
ASR	% angry	0.42
	% correct answers	0.78
	% disgust	0.52
	% fearful	0.13
	% happy	0.50
	% lust	0.68
	% negative emotions	0.73
	% neutral	0.64
	% positive emotions	0.61
	% sad	0.56
Eye tracking–activity monitoring	Activity (ratio)	0.64
	Background (ratio)	0.65
	Body (ratio)	0.63
	Distractors (ratio)	0.64
	Head (ratio)	0.71
	Person (ratio)	0.65
Eye tracking–biodetection	d‐prime (masked condition)	0.59
Eye tracking–biomotion	Latency (ms)	0.68
	Orienting pref (ratio)	−0.10
	Looking pref (ratio)	0.91
Eye tracking–gaze discrimination	Eyes (ratio)	0.12
	Inside face (ratio)	0.75
	Mouth (ratio)	0.80
	Nose (ratio)	0.55
Eye tracking–gender discrimination	Eyes (ratio)	0.59
	Inside face (ratio)	0.75
	Mouth (ratio)	0.68
	Nose (ratio)	0.28
Eye tracking–human activity	Preference (ratio)	0.72
Eye tracking–WAVW	Background (ratio)	0.66
	Body (ratio)	0.80
	Eyes (ratio)	0.71
	Head (ratio)	0.81
	Mouth (ratio)	0.80
Eye tracking–total	Composite score	0.67
Pupillometry	Activity monitoring (mm)	0.94
	Biodetection (mm)	0.96
	Biomotion (mm)	0.96
	Gaze discrimination (mm)	0.93
	Gender discrimination (mm)	0.94
	Human activity (mm)	0.77
	WAVW (mm)	0.96

ADOS: autism diagnostic observation schedule; ASR: affective speech recognition; ICC: intra‐class coefficient of correlation; pref: preference; WAVW: Who's Afraid of Virginia Woolf.

### Pupillometry

Across eye‐tracking paradigms, no consistent significant associations of pupil diameter and ADOS severity or adaptive skills measures were observed (Table 2). However, highly consistent predictions of larger pupil size were seen; across six of the seven paradigms, with lower ABC‐Hyperactivity scores (*r* values ranging from −0.36 to −0.43). Moreover, significant associations between larger pupil diameter and fewer symptoms on the ABC Inappropriate Speech subscale were observed in three of seven paradigms (all *r* = 0.341; *N* = 36; *P* < 0.05). Consistent positive correlations were observed between larger pupil diameters and higher scores in both performance IQ (PIQ) and FSIQ (ranging from *r* = 0.36; *N* = 35; *P* < 0.05 between biological motion detection and PIQ; to *r* = 0.45; *N* = 36; *P* < 0.01 between gender discrimination and FSIQ).

### Olfaction

Olfaction identification was singularly associated with ADOS communication severity (*r* = −0.34; *N* = 38; *P* < 0.05). Olfaction scores were also modestly correlated with VABS adaptive skills measures of Communication, Summary scores, and Adaptive Composite scores (*r* = 0.31; *N* = 38; *P* < 0.1 for all). Better olfactory identification scores were associated with better emotion identification on both RMET (*r* = 0.54; *N* = 38; *P* = 0.0005) and ASR (*r* = 0.40; *N* = 37; *P* = 0.01), and with lower scores on the inappropriate speech subscale of the ABC (*r* = −0.32; *N* = 38; *P* = 0.05). Better olfactory identification was also related to higher FSIQ and VIQ scores (FSIQ: *r* = 0.40; *N* = 38; *P* < 0.05; VIQ *r* = 0.47; *N* = 38; *P* < 0.005).

### Affective Speech Recognition

Higher ASR accuracy was found to be significantly associated with ADOS Stereotyped Behavior (*r* = −0.32; *N* = 37, *P* = 0.05). Better ASR scores predicted higher VABS adaptive skills ratings on the Communication domain (*r* = 0.46; *N* = 37; *P* < 0.005), Summary scores (*r* = 0.40; *N* = 37; *P* < 0.05), and the Adaptive Behavior Composite score (*r* = 0.40; *N* = 37; *P* < 0.05). ASR accuracy was also associated with the two ASD core behavioral symptom dimensions from the ABC. Specifically, with lower scores on the ABC stereotypic behavior (*r* = −0.41; *N* = 37; *P* < 0.05) and the inappropriate speech subscales (*r* = −0.66; *N* = 37; *P* < 0.0001). Better ASR was also associated with better performance on RMET (*r* = 0.64; *N* = 37; *P* < 0.0001). IQ correlated with recognition of affective speech (FSIQ: *r* = 0.54; *N* = 37; *P* < 0.001; VIQ: *r* = 0.45; *N* = 37; *P* < 0.01; PIQ: *r* = 0.43; *N* = 37; *P* < 0.01).

### RMET

This measure of TOM and visual emotion recognition also showed a consistent pattern of significant correlations across ASD clinical dimensions but was not associated with adaptive functioning. In particular, better RMET performance was associated with lower ADOS Total severity (*r* = −0.35; *N* = 38; *P* < 0.05), and ADOS Stereotypic Behavior and Restricted Interests domain (*r* = −0.45; *N* = 38; *P* < 0.005). Regarding associations with behavior, better RMET emotion recognition was seen to predict lower ABC scores on the stereotypic behavior (*r* = −0.33; *N* = 38; *P* < 0.05), hyperactivity (*r* = −0.39; *N* = 38; *P* < 0.05), and inappropriate speech (*r* = −0.50; *N* = 38; *P* < 0.005) subscales. Similar to olfaction and ASR, RMET accuracy was also influenced by IQ (FSIQ: *r* = 0.49; *N* = 38; *P* < 0.005; VIQ: *r* = 0.41; *N* = 38; *P* < 0.01).

### SCIT

A higher total score on the SCIT (indicating better social communication) strongly correlated with core ASD severity measures, but not with adaptive functioning associated behaviors, RMET or ASR, and only showed correlations with two eye‐tracking variables. Higher SCIT total scores predicted lower scores on ADOS total (*r* = −0.61; *N* = 38; *P* < 0.0001), ADOS communication (*r* = −0.45; *N* = 38; *P* = 0.005), reciprocal social interaction (*r* = −0.64; *N* = 38; *P* < 0.0001), and combination of the two subscales (*r* = −0.63; *N* = 38; *P* < 0.0001), with lower CGI‐Severity scores (*r* = −0.51; *N* = 38; *P* < 0.005).

### Test–Retest and Inter‐Rater Reliability

Test–retest reliability analysis (reported in ICC) was carried out for measures performed twice within the same study visit (i.e., ASR and eye tracking). The ICC scores for individual items from the ASR and eye tracking ranged from moderate to high (Table 2).

Approximately 80% of the measures in the eye tracking had good to excellent ICCs [0.63–0.96], with the highest ICC scores observed in pupillometry for all tasks ranging from 0.93 to 0.96 across all measurements except for human activity preference (ICC = 0.77). The reliability of the measurement of individual social AOI proved to be consistent across different paradigms, with higher ICCs detected in looking at the head (0.71–0.81), mouth (0.68–0.80), and body (0.63–0.80), and lower ICCs for looking at the eyes (0.12–0.60) and nose (0.28–0.55). High test–retest reliability was also supported in the ASR percentage of correct answers (ICC = 0.78).

Inter‐rater reliability was calculated for SCIT total scores for a total of 20 interviews for which the participants consented to be videotaped. Agreement was found to be excellent comparing site‐rated to centrally rated scores (*r* = 0.72; *P* = 0.003).

## Discussion

### Relationship of Exploratory Assessments with Clinical and Functional Dimensions

This study sought to test the concurrent validity of exploratory assessments (eye tracking, olfaction, ASR, RMET) and a new assessment of core ASD social communication symptomatology (SCIT) by comparison to standardized clinical and functional measures commonly used in the diagnosis of ASD [Aman et al., 1985; Guy, 1976; Sparrow, 2011]. It also addressed the question of whether the former have the potential for use as biomarkers for social relatedness and communication. We used measures that assessed different stages in the extraction and processing of information relevant for social communication and cognition with eye tracking, a more implicit task assessing initial or “proximal” steps; the sense of olfaction presumably influencing social behavior as an intermediate phase; ASR and RMET, representing higher, integrated levels of effortful processing of socially salient information, and SCIT, as a quantitative endpoint of social communication, the ASD core social behavioral endpoint. Not surprisingly, given the complexity of social functioning, our results are challenging to fully integrate, but represent progress toward understanding the relationships between more proximal measures of social cognition with core domains of ASD severity and associated behaviors.

Exploratory measures showed varying associations across ASD severity, adaptive skills, and behavior. Eye tracking endpoints showed little relationship to adaptive behaviors but correlated with the severity of ASD symptoms and behavior measured by the ADOS and ABC. ASR scores correlated with most adaptive behavior domains, as well as severity. Olfaction predicted visual and auditory emotion recognition and was moderately correlated with the VABS, ABC irritability subscale and VIQ. SCIT scores related moderately to multiple severity domains in the ADOS, and was the only measure not related with IQ. RMET accuracy was less related to ASD features. The correlations which survived Bonferroni correction were those between the RMET and ASR, ASR and the inappropriate speech subscale of the ABC and the correlations between SCIT with the ADOS total and the domains reciprocal social interaction and combined communication and social interaction. Overall, our data suggest that each of the exploratory measures examined have the sensitivity to capture information that individually informs aspects of social functioning, but they appear to largely tap functional differences that are at least partially independent. Their application may depend on the particular domain or function that one intends to interrogate. Our results also suggest that these measures could be refined to increase their sensitivity, given the mostly modest associations found across one “level” of functioning to another. Nevertheless, our data supports further efforts to pursue these as clinical endpoints in studies of novel treatments aimed at reducing core social deficits in ASD.

Measures of more “proximal” steps, that is, performance on multiple eye‐tracking tasks, replicated some but not all extant results from prior studies, which for the most part originated in theoretic studies of mechanism in ASD in toddlers and children. The Activity monitoring, Gender discrimination, and Complex social movies (WAVW) tasks showed the greater number of significant associations with differing ADOS dimensions of ASD severity, and two (Activity monitoring and WAVW) correlated with ADOS Total severity. However, associations were small to moderate.

Notably, eye‐tracking endpoints from our sample of high‐functioning adults with ASD showed little if any relationship to adaptive functioning domains as measured by the VABS. A few studies in high‐functioning adolescents and young adults with ASD have investigated the correlations of the eye tracking with standardized assessments of clinical dimensions. Some of these have found an association between the total fixation times in looking at the mouth and stronger language and social skills in various measures of socialization, ADOS [Klin et al., 2002], VABS [McPartland, Webb, Keehn, & Dawson, 2011; Norbury et al., 2009], Social Responsiveness Scale [Fujioka et al., 2016], and Autism Diagnostic Interview‐Revised (ADI‐R) [Zamzow et al., 2016]. Moreover, correlations between fixation on faces and the overall severity of autism as assessed by the Childhood Autism Rating Scale and the ADI‐R [Grynszpan & Nadel, 2014] have also been described. However, all of these studies were smaller. Although the eye region provides key non‐verbal information for social interactions, individuals with ASD may rely more on decoding verbal information in order to improve the quality of their social exchanges, and therefore would look more to the mouth than to the eyes, other facial features and expressions and body language. Time looking at the mouth was also associated lower STAI totals and higher VIQ. This potentially reflects relationships with social anxiety [Wieser, Pauli, Alpers, & Mühlberger, 2009] and a natural relationship between looking at the mouth for verbal information and appreciation and utility of that information in participants, respectively. Looking inside of the face during the gender discrimination task was also associated with fewer symptoms measured with the inappropriate speech subscale of the ABC. Findings relating eye‐looking to stereotyped behaviors and restricted interests were, however, unexpected, and could point to a potentially inverse association. Future eye tracking analyses could clarify such relationships.

It is important to note that some of the tasks employed in this study were videos of children playing, which may not be the most appropriate or engaging stimuli for adults, and have only been used with toddlers and children in prior research. While age differences between our study sample and previous reports (adults *versus* adolescents) may underlie the differences in results, to our knowledge, our study is the first to illustrate a significant relationship between multiple eye tracking endpoints and IQ. Given that both IQ and age have been identified as factors influencing the severity of ASD symptoms [Charman et al., 2017] and development of compensation in social skills, it is conceivable that people with ASD may apply different strategies to extract socially relevant information at different developmental stages. Further research on this subject is needed to identify the most suitable biomarkers for each stage of the developmental trajectory [Beversdorf Missouri Autism Summit, 2016; Thompson & Levitt, 2010]. Notwithstanding these differences, dynamic social tasks such as Activity monitoring and WAVW tasks, and the AOI time spent looking at the mouth, appear to be an adequate biomarker for measuring ASD severity, providing some context on the socialization ability in adults with ASD, while apparently being less influenced by daily living skills, a possible proxy of treatment, and non‐core associated behaviors.

Pupillometry measures were mostly unrelated to ASD severity and core social deficits. However, larger pupil size was consistently related to lower behavioral ratings of hyperactivity. As a reflection of greater arousal or effort while engaged in task performance, pupil size may indicate greater cognitive or inhibitory control and prove its utility in studying this separate important dimension of co‐occurring inattentive and disruptive behavior symptoms in ASD [McCracken, 2011]. Similarly, the association of IQ with pupil size may reflect the ability to better marshal effortful attention during the eye tracking.

Olfaction is known to play an important role in social communication in rodents and potentially in humans [Purves et al., 2001; Hays et al., 2003; Wysocki et al., 2004]. Despite sparse attention in ASD, Rosenkrantz et al. showed that altered olfaction may contribute to abnormal processing of socially salient information and/or provide a biomarker indexing disruptions of the embryogenic development within critical time‐frames [Rosenkrantz et al., 2015]. In our study, olfaction showed significant association with ADOS Communication severity. The correlation of olfactory accuracy with visual and auditory emotion identification (RMET; ASR), supports the notion of cross‐modal sensory processing impairments in ASD. These strong links between olfaction and the development of social cognition, suggest that olfaction may be a useful biomarker for social and functioning deficits in ASD in examining the trajectory of this developmental ability.

At the same time, ASR lacked discrete associations with clinical severity ratings of ASD communication and social reciprocity, but it was the only measure that significantly correlated with VABS Communication and the Adaptive Behavior Composite score. The ASR was also significantly negatively associated with stereotypic behavior, both from the ADOS and the ABC subscale, which possibly points to mechanistic links between this social cognitive ability and ASD behavior that are currently unknown. Given these associations, the use of the ASR as a stratification variable in treatment trials is of interest, as well as its possible ability to index short‐term changes that may ultimately predict changes in adaptive skills.

The RMET, also an emotion recognition task, showed several parallel associations with ASR, except that it did not predict adaptive behavior skills. Similar to the ASR, RMET accuracy was related to stereotypic behavior on ADOS and ABC measures as well as to the ADOS Total severity.

The moderate to large correlations between the SCIT total scores with most ADOS scores, and better CGI‐S ratings provides solid evidence that it performs well as a relatively concise metric of social communication impairment in ASD. Somewhat surprisingly, SCIT scores were relatively independent of adaptive skills measures and showed no significant relationship to IQ or maladaptive behaviors. If VABS scores reflect cumulative effects of intervention, it could be argued that the SCIT may be useful in shorter‐term intervention trials aiming to capture a more integrated assessment of changes in the core social functioning in ASD, although this remains to be established.

### Feasibility

Our study demonstrates the feasibility of implementation of these assessments in a clinical study setting as measured by the large amount of completed assessments. We found moderate‐to‐high ICCs in ASR and across all the eye‐tracking tasks, and very good inter‐rater reliability on the SCIT, supporting the application of these assessments in multi‐site treatment studies. Operationally, the SCIT, ASR, RMET, and olfaction test do not require extensive infrastructure and are therefore suitable for multicenter studies. However, the cultural adaptability of the ASR depends on developing validated translated versions to extend its application internationally. The use of eye tracking is limited by non‐standardized data processing, however, as an implicit performance measure [Emery, 2000] it has greater utility for assessment across the lifespan and level of functioning in ASD while the ASR and RMET are likely to be more suited to higher functioning patients; at present, validity for lower‐functioning and different cultural samples remain unclear.

### Limitations

Our findings are limited by a small sample size of all‐male, high‐functioning adults; generalizability to female and low‐functioning adult patients with ASD has to be established. The fact that subjects in Study 2 had to score ≤13 on ABC irritability and undergo a drug intravenous infusion for 2 h, may have selected participants with less anxiety and disruptive behaviors than a broader ASD population and the population in Study 1 (STAI total score: Study 1 = 44.8 *vs* Study 2 = 32.6; *P* = 0.004; ES = 1.0). More broadly, it would be of interest to examine how the observed biomarkers may extend to those with more profound social impairments, intellectual disability, and other disorders. In our study, although we did not control for previous treatment history, we limited the effects of concomitant psychotherapy on the cognitive and behavioral assessments by the inclusion of patients who were on stable existing medication and not allowing medication adjustments during the study. The overall proportion of patients receiving psychotropic medications in our study is lower than what was found in the research by Houghton et al. [2017] in a database of over 90,000 children and adults with ASD. Eye‐tracking tasks contained multiple, embedded experimental conditions with pre‐specified hypotheses that were not modeled in current statistical analyses. Including these condition‐specific effects could reduce variance and reveal further, more robust associations with clinical phenotype.

Test–retest reliability of outcomes was measured over a short period; therefore, the estimated intra‐class correlations are likely to be positively biased. Finally, it should be noted that treatment effects on biomarkers used as surrogate outcomes do not always predict true clinical outcomes. Therefore, the actual applicability of a biomarker to predict drug response would need to be tested and validated in an interventional clinical study.

## Conclusions

This work aimed to establish the validity of exploratory behavioral measures of social communication (SCIT, ASR, RMET, olfaction, and eye tracking) in adults with high‐functioning ASD. More explicit measures of higher‐order processes (i.e., SCIT, ASR, RMET, and olfaction identification) showed a convincing convergence with clinical and functioning measures of ASD making them acceptable measures of deficits in social communication and cognition. However, more “proximal” and implicit behavioral biomarkers (i.e., eye tracking) showed fewer, albeit consistent and plausible, task‐based associations with clinical symptomatology. The data suggest that these measures capture processes that are partially independent but still inter‐related, and that each provides unique information across a range of domains of social processing and behavior. To guarantee reliable results mapping onto clinical symptomatology, further refinement and specificity in the selection of eye‐tracking paradigms and AOIs are crucial. Measurements of the performance during paradigms, such as the activity‐monitoring, WAVW, gender discrimination; and the area of interest looking at the mouth proved to provide more information on ASD severity. However, due to the small sample size and the exploratory nature of this work, further research is needed to confirm the use of these measures as surrogates in clinical trials and to establish reliable biomarkers of social and communication deficits in adults with ASD. The selection of one or another measure in future interventional clinical trials will depend largely on the mechanism of action of the drug.

## Conflicts of interest

E.H. has provided consultation to Roche and received research grants from Roche, Curemark, Coronado Biosciences, Forest, Simons Foundation, Foundation for Prader Willi Research, and the Orphan Products Division of the Food and Drug Administration, and has intellectual property relating to oxytocin and autism. F.S. has provided consultation to Roche and Janssen Pharmaceutical and has received research grants from Roche, NIH, and the Simons Foundation. J.T.M. has served as a consultant for Roche and Dart Neuroscience, has received research grants from Roche, and has received study drug from Shire and AstraZeneca. F.S. has received research funding from Roche and Janssen Pharmaceuticals. M.V.R., D.U., J.N., L.B., O.K., S.S., and P.F. are full‐time employees of F. Hoffmann‐La Roche.

## Data sharing statement

Qualified researchers may request access to individual patient level data through the clinical study data request platform (http://www.clinicalstudydatarequest.com/). Further details on Roche's criteria for eligible studies are available here (https://clinicalstudydatarequest.com/Study-Sponsors/Study-Sponsors-Roche.aspx). For further details on Roche's Global Policy on the Sharing of Clinical Information and how to request access to related clinical study documents, see here (https://www.roche.com/research_and_development/who_we_are_how_we_work/clinical_trials/our_commitment_to_data_sharing.htm)).

## Supporting information


**Appendix S1:** Supplementary Material
