# Supplementary material for: In Search of Biomarkers for Autism Spectrum Disorder
Source: Autism Res. 2018 Oct 15;11(11):1567–79. doi: 10.1002/aur.2026 (PMC6282609; doi:10.1002/aur.2026)
Supplement: Supplementary file 1 — Appendix S1: Supplementary Material [file AUR-11-1567-s001.docx]

**In search of biomarkers for Autism Spectrum Disorder**

**Authors:** Marta del Valle Rubido^1^, James T. McCracken^2^, Eric Hollander^3^, Frederick Shic^4,5^, Jana Noeldeke^1^, Lauren Boak^6^, Omar Khwaja^1^, Shamil Sadikhov^1^, Paulo Fontoura^6^ and Daniel Umbricht^1^

1. Roche Pharmaceutical Research and Early Development NORD, Roche Innovation Center, Basel, Switzerland

2. Psychiatry and Behavioral Sciences, David Geffen School of Medicine at UCLA, Los Angeles, USA

3. Psychiatry and Behavioral Sciences, Albert Einstein College of Medicine and Montefiore Medical Center, Bronx, New York, USA

4. Center for Child Health, Behavior and Development, Seattle Children's Research Institute, Seattle, WA, USA;

5. Department of Pediatrics, University of Washington, Seattle, WA, USA

6. Roche Product Development Neuroscience, Basel, Switzerland

**Corresponding author**: Marta del Valle Rubido, Roche Pharmaceutical Research and Early Development NORD, Roche Innovation Center, Basel, Switzerland

Telephone: +41 616877557; Fax: +41 616889848; Email: marta.del_valle_rubido@roche.com

**Supplementary Table 1.** Inclusion and exclusion criteria

| **Study 1 (individuals with autistic disorder or Asperger’s syndrome)** | | **Study 2** | |
| --- | --- | --- | --- |
| **Inclusion criteria** | **Exclusion criteria** | **Inclusion criteria** | **Exclusion criteria** |
| Individuals with a diagnosis of autistic disorder or Asperger’s syndrome as defined by DSM-IV-TR, confirmed by the site’s clinical team and supported by the ADOS | – | Individuals with a diagnosis of autistic disorder as defined by DSM-IV, confirmed by the site’s clinical team and supported by the ADOS | Unwilling to use an effective form of contraception as deemed appropriate to individual patient by the investigator (for example abstinence or condoms) for the duration of the study and for at least 7 days after the last dose |
| Male adults aged 18–45 years (inclusive) | Positive test for drugs of abuse or alcohol | Male adults (age 18–45 years) | Positive urine test for drugs of abuse |
| IQ >70 | Alcohol and/or substance abuse/dependence during the last 12 months | IQ >70 (WASI) | Alcohol and/or substance abuse/dependence during the last 12 months |
| BMI 18–35 kg/m^2^ (inclusive) | – | BMI 18–35 kg/m^2^ (inclusive) | A current (at screening) significant risk of suicidal behavior as judged by the investigator following a thorough clinical evaluation and supported by information collected on the C-SSRS |
| – | – | ABC – irritability subscale score ≤13 | Positive result on hepatitis B, hepatitis C, or human immunodeficiency virus 1 and 2 |
| Language, hearing and vision compatible with the study measurements as judged by the investigator | Confirmed (e.g., two consecutive measurements) SBP >140 mmHg or <90 mmHg, and DBP >90 mmHg or <50 mmHg | Language, hearing, and vision compatible with the study measurements as judged by the investigator | Confirmed SBP >140 mmHg or <90 mmHg, and DBP >90 mmHg or <50 mmHg |
| Existing medication regimens were stable for an appropriate duration as deemed by the investigator prior to day 1 | Resting PR >100 bpm or <40 bpm | Existing medication regimens should be stable for 4 weeks, with the intent to remain stable throughout the study | Confirmed resting PR >100 bpm or <45 bpm |
| Able to participate and willing to give written informed consent and to comply with the study restrictions | – | Able to participate and willing to give written informed consent and to comply with the study restrictions | Confirmed clinically significant abnormality on 12-lead electrocardiogram, including a QT of ≥500 milliseconds |
| The availability of a reliable caregiver, able and willing to provide information regarding the individual’s behavior and symptoms | – | The presence of a reliable caregiver, able and willing to provide information regarding the individual’s behavior and symptoms | Clinically significant abnormalities in laboratory test results (including hepatic and renal panels, complete blood count, chemistry and coagulation panels, and urinalysis) |
|  | – |  | Active stomach ulcer disease, history of coagulopathies or bleeding disorders |
|  | – |  | Active inflammatory pulmonary disease |
|  | History of epilepsy/seizure disorder (except simple febrile seizures) |  | History of epilepsy/seizure disorder (except simple febrile seizures) |
|  | Significant disruptive, aggressive or self-injurious, or sexually inappropriate behavior during the last 3 months that, in the opinion of the investigator, might interfere with the conduct of the study |  | Significant disruptive, aggressive or self-injurious, or sexually inappropriate behavior during the last 3 months that, in the opinion of the investigator, might interfere with the conduct of the study |
|  | Concomitant disease or condition that could interfere with, or treatment of which might interfere with, the conduct of the study, or that would, in the opinion of the investigator, pose an unacceptable risk to the subject in this study |  | Any significant uncontrolled or any unstable medical condition other than autistic disorder (e.g., diabetes) that might interfere with the conduct of the study, confounds interpretation of the study results, or endangers the patient’s well-being |
|  | – |  | Initiation of new or major change in psychosocial intervention within 4 weeks prior to randomization. Minor changes in ongoing treatment (e.g., missed therapy sessions due to holiday/vacation; planned break in therapy due to school holidays; changes in college/school programs) are not considered significant |
|  | – |  | Currently receiving treatment with prohibited medications and not willing to cease treatment for the minimum time period before randomization |
|  | – |  | Treatment with any investigational agent within 90 days prior to screening |
|  | Donation of blood >500 mL within 3 months prior to screening |  | Donation of blood >500 mL within 3 months prior to screening |
|  | History of hypersensitivity or allergic reactions |  | History of hypersensitivity or allergic reactions |
|  | Lack of peripheral venous access |  | Lack of peripheral venous access |

ABC, Aberrant Behavior Checklist; ADOS, Autism Diagnostic Observation Schedule; BMI, body mass index; bpm, beats per minute; C-SSRS, Columbia-Suicide Severity Rating Scale; DBP, diastolic blood pressure; DSM-IV-TR, Diagnostic and Statistical Manual of Mental Disorders, 4th Edition, Text Revision; PR, pulse rate; SBP, systolic blood pressure; WASI, Wechsler Abbreviated Scale of Intelligence.

**Supplementary Table 2.** Baseline characteristics of ASD patients in Studies 1 and 2

| **Variable** | **ASD current study participants Mean (SD), *N* = 19** | **ASD interventional study participants Mean (SD), *N* = 19** |
| --- | --- | --- |
| Age in years | 25.9 (5.48) | 23.4 (5.16) |
| Median age in years  and range | 26  (19-39) | 22  (18-40) |
| Male | 19 | 19 |
| Race, *N*  White  Asian  Black/African–American  Native Hawaiian/Pacific Islander  Other | 16 (89%)  1 (6%)  0  0  1 (6%) | 17 (94%)  0  0  1 (6%)  0 |
| WASI – full-scale IQ | 102.3 (14.31) | 99.9 (14.55) |
| WASI – verbal IQ | 103.2 (14.57) | 97.8 (17.41) |
| WASI – performance IQ | 100.5 (14.50) | 100.7 (11.09) |
| ADOS – communication | 3.0 (0.7) | 3.0 (1.7) |
| ADOS – social interaction | 7.0 (1.8) | 6.0 (2.4) |
| ADOS – communication and social interaction | 10 (2.2) | 10 (3.7) |
| ADOS total | 12 (2.5) | 12 (4.8) |
| Vineland-II – adaptive behavior composite | 64 (11.0) | 63 (12.5) |
| Vineland-II – communication | 64 (14.0) | 65 (21.8) |
| Vineland-II – daily living | 72 (13.4) | 66 (9.6) |
| Vineland-II – socialization | 64 (13.5) | 65 (13.0) |
| ABC total | 39 (20.8) | 27 (19.9) |
| ABC – irritability | 8 (7.7) | 3 (4.0) |
| ABC – lethargy/social withdrawal | 14 (7.2) | 9 (6.7) |
| ABC – stereotypic behavior | 4 (4.5) | 3 (2.6) |
| ABC – hyperactivity | 9 (7.0) | 9 (9.3) |
| ABC – inappropriate speech | 3 (2.7) | 3 (2.7) |
| STAI | 44 (15.1) | 33 (8.3) |

ABC, Aberrant Behavior Checklist; ADOS, Autism Diagnostic Observation Schedule; ASD, Autism Spectrum Disorder; STAI, State/Trait Anxiety Inventory-State; WASI, Wechsler Abbreviated Scale of Intelligence.

**Supplementary Materials 1.** Prescription patterns of psychotropic medication at baseline in patients with ASD.

The prescription pattern of psychotropic medications was evaluated. 55.3% of the ASD patients had at least one prescription. Nine patients (23.7%) used two drugs, five patients (13.2%) used one drug; three patients used three, three patients used four (7.9%) and one patient (2.63%) used five psychotropic medications. The classes of psychotropic medications most frequently used in our study are antidepressants/SSRI/SNRI (12 prescriptions) followed by antipsychotic and antimanic agents (11 prescriptions), anticonvulsants (nine prescriptions) and anorexiants and CNS stimulants (five prescriptions).

**Supplementary Materials 2.** Details of clinical and functional assessments, eye-tracking paradigms and pupillometry

**The Wechsler Abbreviated Scale of Intelligence version II (WASI-II).**The WASI-II [Wechsler, 2008] is a measure of adult intelligence providing three primary estimates of intelligence quotients (IQ): verbal IQ (VIQ), performance IQ (PIQ), and full-scale (FSIQ).

**Autism Diagnostic Observation Schedule module 4 (ADOS).** The ADOS [Lord et al., 2002] is a validated examiner-rated instrument that systematically prompts assessment of social behavior and interaction for assessment of ASD diagnoses. The assessment scores the domains of social communication, social relatedness, imagination, and restricted/repetitive behaviors. Three domains are used for determining standard cutoff scores for ASD diagnosis: communication, social interaction, and the combined communication and social interaction score. Higher scores indicate greater levels of core deficits.

**Aberrant Behavior Checklist – Community Version (ABC-C).**The ABC-C [Aman et al., 1985] is a -item, informant-rated questionnaire that rates individuals across the following factors over the past four weeks: irritability, lethargy and social withdrawal, stereotypic behavior, hyperactivity/non-compliance, and inappropriate speech over the past four weeks. Items are scored from 0 (no problem) to 3 (severe problem).

**Vineland Adaptive Behavior Scale-II (VABS-II.** The Vineland-II [Sparrow, 2011] is a caregiver clinician interview for assessment of adaptive behavior in intellectual and developmental disorders. It measures adaptive behavior across the domains such as communication, daily living skills, and socialization. A composite score is given as a measure of overall functioning. The interviewer asks general questions pertaining to the subjects functioning in each subdomain and rates the examinee on critical behaviors (2: always present, 1: sometimes present, 0: seldom or never present). Raw scores are converted to age equivalent standard scores for each domain and for the composite adaptive behavior score. Higher scores indicate greater levels of adaptive functioning.

**Clinical Global Impression-Severity (CGI-S).**The CGI-S [Guy, 1976] is a clinician-rated measure of the severity of illness. Ratings are made on a seven-point scale ranging from 1 (normal) to 7 (among the most extremely ill).

**State/Trait Anxiety Inventory-State (STAI).** The STAI [Spielberger, 2010] is a measure of state anxiety with 20 self-completed items. Items are scored on a Likert scale of 1 (not at all) to 4 (very much so). Higher scores in the STAI indicate the presence of worse symptoms.

**Eye tracking.** Eye tracking is a technique used to investigate patterns of scanning and gaze behavior and the nature of the information used by a subject to process stimuli and extract socially salient data from complex visual scenes. Eye tracking has emerged as an important tool to detect differences in gaze behavior and processing of socially salient stimuli between healthy participants and individuals with ASD. A Tobii T60XL 60 Hz eye-tracking system was used at all sites to continually measure gaze position and pupil size during the following paradigms:

(1) Activity Monitoring [Shic et al., 2011]: subjects are played a recording of two human actors performing a social activity (simple play activities) with visually salient distracters in the background. Dependent variables include attention (% of total fixation time) to 1) either of the two people in the scene; 2) the area of shared focus/central activity between the two actors in the video clips; and 3) background elements including distractors.

(2) Biological motion preference task (biomotion) [Kaiser et al., 2010]: subjects watch two side-by-side videos, in random left-right order, each containing dynamic point-light displays; one video is derived from video recordings of a human actor’s performance of an activity such as walking, jumping a rope, or waving. The other video is a computer-generated animation of moving dots. Dependent variables include attention (total fixation time) to video 1 versus video 2, and orienting to video 1 versus video 2.

(3) Biological motion detection (biodetection): subjects are shown a video of scrambled and unscrambled point light displays of human walkers, and computer-generated animation of moving dots under masked and unmasked conditions. Participants are asked to identify whether it is a human being walking or not and the dependent variable calculated was d-prime, signal detection measurement on masked and unmasked human and non-human motion.

(4) Complex social tasks: subjects are shown a short clip of the movie ‘Who’s Afraid of Virginia Woolf” (WAVW) [Klin et al., 2002], which displays complex social interactions with a high emotional load between two human characters. Dependent variables include attention (% of total fixation time) to 1) eyes of the actors; 2) mouths of the actors; 3) the bodies of the actors; and 4) background regions including objects.

(5) Gaze discrimination and (6) gender discrimination in a static face-scanning task [Andari et al., 2010], where each participant views a series of static photographs of human faces with instructions tapping into specific cognitive faculties: report the gaze direction (direct/averted) and the gender (male/female) of the person shown in the photograph. Primary dependent variables include attention (total fixation time) to 1) facial regions and 2) non-facial regions and secondary dependent variables include time spent looking at the mouth and eyes of the presented faces.

(7) Human activity preference task (human activity or social vs geometric) [Pierce et al., 2011], two videos are presented simultaneously, side by side, on a single screen. One video presents a recording of a human performing an activity, such as walking or shoveling sand, while the other shows a computer-generated, continuously moving or changing geometric pattern. The left–-right ordering of videos is counter-balanced for each series and participant. Dependent variable is the attention (total fixation time) to video 1 versus video 2.

Trials were considered valid if they contained more than 70% valid eye tracking data and exhibited a calibration uncertainty of less than 1.5 degrees in the trial or less than 3 degrees over the entire session [Shic, 2008]. A participant's session data was considered valid for a paradigm if it contained 50% or more valid trials.

**Pupillometry.** Dysregulated tonic pupil size during social interaction has been reported as a behavioral feature of ASD [Corbett et al., 2010]. Pupillometry is used to measure changes in pupil diameter as an index of autonomic arousal during the performance of the eye-tracking paradigms and this measurement is integrated into the eye-tracking apparatus [Anderson et al., 2013]. In our study, we did not consider dynamic changes, only tonic pupil sizes.

**Supplementary Materials 3.** Composite eye-tracking score calculation

The full eye-tracking composite, C_TOT_, is computed by taking into account contributions from individual experiments: activity monitoring (C_AM_ = %Activity + %Head), biomotion detection (C_BD_ = d´), biological motion preference (C_BP_ = %Biomotion looking time preference + 0.5 %Biomotion orienting preference), face scanning (C_FS_ = (%Eyes + %Mouth)_Gender Discrimination Task_  + (%Eyes + %Mouth)_Gaze Discrimination Task_), human activity (i.e. social geometry, C_SG_ = %Social Preference), and complex naturalistic social movies (i.e., Who's Afraid of Virginia Woolf?, C_WAVW_ = %Eyes + %Mouth). Each sub-composite value of C_TOT_ was computed by applying, to all data associated with the sub-composite experiment, a z-score transformation based on means and standard deviations from the first session, i.e., for session *s* and experiment *X*, $Z_{X}^{s}=\frac{C_{X}^{S}-\mu\left[ C_{X}^{1} \right]}{\sigma\left[ C_{X}^{1} \right]}$ and by making use of preserved sign square and square root functions, ${sgn}^{2}\left( x \right)=x\left| x \right|=sgn\left( x \right)x^{2}, {sgn}^{\frac{1}{2}}\left( x \right)=sgn\left( x \right)\sqrt{\left| x \right|}$

$C_{TOT}^{S}={sgn}^{\frac{1}{2}}\left( {0.2sgn}^{2}\left( Z_{AM}^{s} \right)+{0.1sgn}^{2}\left( Z_{BD}^{s} \right)+{0.2sgn}^{2}\left( Z_{BP}^{s} \right)+{0.2sgn}^{2}\left( Z_{FS}^{s} \right)+{0.15sgn}^{2}\left( Z_{SG}^{s} \right)+{0.15sgn}^{2}\left( Z_{WAVW}^{s} \right) \right)$

**References**

Aman, M. G., Singh, N. N., Stewart, A. W., & Field, C. J. (1985). Psychometric characteristics of the aberrant behavior checklist. American Journal of Mental Deficiency 89, 492–502.

Andari, E., Duhamel, J.-R., Zalla, T., Herbrecht, E., Leboyer, M., & Sirigu, A. (2010). Promoting social behavior with oxytocin in high-functioning autism spectrum disorders. Proceedings of the National Academy of Sciences 107, 4389–4394.

Anderson, C. J., Colombo, J., & Unruh, K. E. (2013). Pupil and salivary indicators of autonomic dysfunction in autism spectrum disorder. Developmental Psychobiology 55, 465–482.

Corbett, B. A., Schupp, C. W., Simon, D., Ryan, N., & Mendoza, S. (2010). Elevated cortisol during play is associated with age and social engagement in children with autism. Molecular Autism 1, 13.

Guy, W. (1976). ECDEU assessment manual for psychopharmacology: 1976. National Institute of Mental Health.

Kaiser, M. D., Delmolino, L., Tanaka, J. W., &. Shiffrar, M. (2010). Comparison of visual sensitivity to human and object motion in autism spectrum disorder. Autism Research 3, 191–195.

Klin, A., Jones, W., Schultz, R., Volkmar, F., & Cohen, D. (2002). Visual fixation patterns during viewing of naturalistic social situations as predictors of social competence in individuals with autism. Arch Gen Psychiatry 59, 809–816.

Lord, C., Rutter, M., DiLavore, P., & Risi, S. (2002). Autism diagnostic observation schedule: ADOS, Western Psychological Services Los Angeles, CA.

Pierce, K., Conant, D., Hazin, R., Stoner, R., & Desmond, J. (2011). Preference for geometric patterns early in life as a risk factor for autism. Archives of General Psychiatry 68, 101–109.

Shic, F. (2008). Computational Methods for Eye-Tracking Analysis: Applications to Autism (Ph.D. thesis). Yale University.

Shic, F., Bradshaw, J. , Klin, A., Scassellati, B., & Chawarska, K. (2011). Limited activity monitoring in toddlers with autism spectrum disorder. Brain Research 1380, 246–254.

Sparrow, S. (2011). Vineland adaptive behavior scales. Encyclopedia of Clinical Neuropsychology. J. Kreutzer, J. DeLuca & B. Caplan. New York: Springer, p. 2618–2621.

Spielberger, C. D. (2010). State‐trait anxiety inventory. John Wiley & Sons, Inc..

Wechsler, D. (2008). Wechsler adult intelligence scale–Fourth Edition (WAIS–IV). San Antonio, TX: NCS Pearson.
